# ALTernative Functions for Human FANCM at Telomeres

**DOI:** 10.3389/fmolb.2019.00084

**Published:** 2019-09-06

**Authors:** Beatriz Domingues-Silva, Bruno Silva, Claus M. Azzalin

**Affiliations:** Instituto de Medicina Molecular João Lobo Antunes (iMM), Faculdade de Medicina da Universidade de Lisboa, Lisbon, Portugal

**Keywords:** FANCM, telomeres, ALT, R-loops, TERRA, BLM helicase

## Abstract

The human FANCM ATPase/translocase is involved in various cellular pathways including DNA damage repair, replication fork remodeling and R-loop resolution. Recently, reports from three independent laboratories have disclosed a previously unappreciated role for FANCM in telomerase-negative human cancer cells that maintain their telomeres through the Alternative Lengthening of Telomeres (ALT) pathway. In ALT cells, FANCM limits telomeric replication stress and damage, and, in turn, ALT activity by suppressing accumulation of telomeric R-loops and by regulating the action of the BLM helicase. As a consequence, FANCM inactivation leads to exaggerated ALT activity and ultimately cell death. The studies reviewed here not only unveil a novel function for human FANCM, but also point to this enzyme as a promising target for anti-ALT cancer therapy.

## FANCM

Human FANConi anemia, complementation group M (FANCM) is a highly conserved protein with ATPase and DNA translocase activity, belonging to the Fanconi anemia (FA) core complex (Meetei et al., 2005). FA is a hereditary disorder characterized by bone marrow failure, hypersensitivity to agents inducing DNA interstrand crosslinks (ICLs), chromosomal abnormalities and, later in life, cancer. Although FANCM is part of the FA complex, FANCM mutations are not causative of FA (Singh et al., 2009; Bogliolo et al., 2018; Catucci et al., 2018). Nonetheless, some *FANCM* mutations are associated with higher risk for breast and ovarian carcinomas; hence, this enzyme can be considered a tumor-suppressor (Catucci et al., 2018; Nurmi et al., 2019; Schubert et al., 2019).

**Figure 1:**
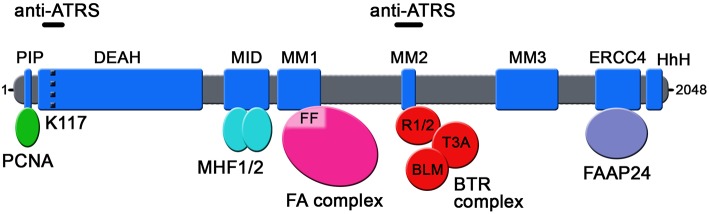
Schematic representation of the domains so far identified in human FANCM protein. The position of lysine 117 (K117) within the ATPase pocket is indicated by a dotted black line. FF: FANCF; R1/2: RMI1 and RMI2; T3A: TOP3A; anti-ATRS: regions identified as necessary for FANCM function in suppressing ALT-associated telomeric replication stress.

Seven independent domains with separable functions have been identified in FANCM so far (Figure 1): (i) the N-terminal PIP-box (aa 5-12), which interacts with proliferating cell nuclear antigen (PCNA) (Rohleder et al., 2016); (ii) the DEAD/DEAH-motif (aa 77-590), with ATPase activity (Meetei et al., 2005) (iii) the MID-motif (aa 661-800), which interacts with the Major Histone Fold 1 and 2 (MHF1/2) heterotetramer (Yan et al., 2010); (iv) the MM1-motif (aa 826-967), which interacts with FANCF within the FA core complex (Deans and West, 2009); (v) the MM2-motif (aa 1218-1251), which interacts with RecQ-Mediated genome Instability protein 1 (RMI1), a component of the so-called BTR complex together with Bloom (BLM) and Topoisomerase IIIA (TOP3A) (Deans and West, 2009; Hoadley et al., 2012); (vi) the ERCC4-motif (aa 1818-1956), which is required for FANCM heterodimerization with its obligatory partner Fanconi Anemia core complex-Associated Protein 24 (FAAP24) (Ciccia et al., 2007); and (vii) the C-terminal HhH domain (aa 1971-2030), which equips FANCM with DNA binding activity (Coulthard et al., 2013; Yang et al., 2013). FANCM also comprises the MM3 domain (aa 1502-1708; Figure 1) of still unknown function (Deans and West, 2009).

FANCM association with the FA complex promptly suggested a role in the repair of ICL lesions (Meetei et al., 2005). Indeed, when a replication fork encounters an ICL, the FANCM-FAAP24-MHF1-MHF2 complex enhances the recruitment of the FA complex through interaction between FANCM MM1 and FANCF (Deans and West, 2009). This stimulates the monoubiquitination of FANCD2, another FA component, an essential event for ICL disengagement and DNA damage repair (Meetei et al., 2005; Mosedale et al., 2005; Yamamoto et al., 2011; Klein Douwel et al., 2014). However, in absence of FANCM, the FA complex still monoubiquitinates FANCD2, albeit less efficiently, and triggers repair (Bakker et al., 2009; Singh et al., 2009). This might explain why mutations in the *FANCM* gene are not causative of FA. FANCM also allows remodeling of arrested replication forks and traversing of ICL lesions independently of the FA complex (Huang et al., 2013). This requires FANCM ATPase activity and the interaction of the PIP-motif with PCNA (Huang et al., 2013; Rohleder et al., 2016).

FANCM promotes resolution of genome-wide spread R-loops (Schwab et al., 2015). The replication machinery might stall upon encountering R-loops and a lack of timely resolution of these structures can lead to genome instability (Crossley et al., 2019). In FANCM-deficient cells, R-loops accumulate across the genome and recombinant FANCM unwinds RNA:DNA hybrids in the presence of FAAP24 and ATP (Schwab et al., 2015). An ATPase-inactive mutant of FANCM (FANCM K117R) fails to suppress RNA:DNA hybrids both *in vitro* and *in vivo*, highlighting the importance of FANCM enzymatic activity in resolving R-loops (Schwab et al., 2015; Silva et al., 2019). FANCM involvement in R-loop metabolism appears to be evolutionarily conserved since budding yeasts deficient for the FANCM ortholog Mph1 accumulate genomic R-loops (Lafuente-Barquero et al., 2017). Notably, the FA components FANCD2, FANCA and FANCL also suppress R-loops in human and murine cells (Garcia-Rubio et al., 2015; Schwab et al., 2015). However, since FANCM ATPase activity is dispensable for FA complex function (Xue et al., 2008), FANCM and the other FA factors are likely to avert R-loops through separate mechanisms. FANCM ATPase activity also supports full activation of the ATR checkpoint cascade and common fragile site stability (Collis et al., 2008; Schwab et al., 2010; Wang et al., 2018).

FANCM interaction with RMI1 through its MM2-motif facilitates recruitment of the BTR complex to DNA lesions (Deans and West, 2009). Consistently, FANCM is required for the formation of BLM foci upon treatment with mitomycin C and camptopthecin (Deans and West, 2009). The BTR complex, also named “dissolvasome,” promotes Holliday Junction branch migration and the dissolution of recombination intermediates that could lead to harmful sister chromatid exchange (SCE) events (Karow et al., 2000; Wu and Hickson, 2003). As a consequence, FANCM depletion in human cells causes SCE accumulation, a feature shared with BLM-deficient cells (Neff et al., 1999; Deans and West, 2009).

## Alternative Lengthening of Telomeres

The ends of linear eukaryotic chromosomes, the telomeres, are actively transcribed heterochromatic nucleoprotein structures comprising repetitive DNA sequences (5′-TTAGGG-3′/5′-CCCTAA-3′ in vertebrates), shelterin proteins and the long noncoding RNA TERRA (Azzalin and Lingner, 2015; Shay and Wright, 2019). The inability of canonical DNA polymerases to fully replicate linear DNA molecules at each round of cell division causes progressive telomere shortening, which cannot be buffered in cells lacking mechanisms of *de novo* synthesis of telomeric DNA (Shay and Wright, 2019). Upon extensive shortening, critically short telomeres accumulate in cells and emanate an irreversible DNA damage signal causing permanent growth arrest and eventually cell death (Harley et al., 1990; Nassour et al., 2019). To gain unlimited replicative potential, 85–90% of human cancer cells reactivate the reverse transcriptase telomerase, which utilizes an associated RNA moiety to produce telomeric DNA (Kim et al., 1994; Shay and Bacchetti, 1997). The remaining 10–15% of human cancers elongate telomeres trough homology-directed repair (HDR) pathways collectively known as Alternative Lengthening of Telomeres or ALT (Apte and Cooper, 2017). ALT can thus be considered a specialized DNA repair mechanism assuring cell immortality. ALT was first described in budding yeast survivors arising after crisis induced by telomerase inactivation (Lundblad and Blackburn, 1993). Few years later, ALT was reported in human cells (Bryan et al., 1995, 1997; Dunham et al., 2000). Human ALT cancers are generally of mesenchymal or epithelial origin, and comprise among others some osteosarcomas, liposarcomas, glioblastomas and astrocytomas.

Besides being immortal and telomerase-negative, a number of features characterize ALT cells, including abundant extrachromosomal double-stranded (ds) or single-stranded (ss) telomeric DNA in circular or linear form (Ogino et al., 1998; Tokutake et al., 1998; Cesare and Griffith, 2004; Wang et al., 2004), and specialized nuclear bodies referred to as ALT-associated PML bodies (APBs). APBs contain ProMyelocytic Leukemia (PML), telomeric DNA, TERRA, shelterin components including TRF1 and TRF2, and DNA damage signaling and repair factors including RPA, RAD51, RAD52, BRCA1, and BLM and WRN helicases (Yeager et al., 1999; Johnson et al., 2001; Stavropoulos et al., 2002; Acharya et al., 2014; Arora et al., 2014; Pan et al., 2017). ALT cells are also characterized by elevated rates of exchange of DNA between sister telomeres (T-SCE) and increased transcription of TERRA, likely due to TERRA promoter hypomethylation (Bailey et al., 2004; Lovejoy et al., 2012; Arora et al., 2014). Finally, inactivation of one or both of the ATP-dependent chromatin remodelers Alpha-Thalassemia/mental Retardation X-linked (ATRX) and Death domain-Associated protein-6 (DAXX) are often found in ALT tumors (Heaphy et al., 2011; Lovejoy et al., 2012; Schwartzentruber et al., 2012). ATRX and DAXX form a complex that deposits the histone variant H3.3 at heterochromatic loci, including telomeres. Lack of ATRX and/or DAXX activity may explain the altered chromatin state of ALT telomeres, and possibly the deregulation in TERRA transcription and T-SCEs (Episkopou et al., 2014; Dyer et al., 2017). A recent report revealed that ALT telomeres are enriched in the heterochromatin mark H3 trimethylated at lysine 9 (H3K9me3), deposited by the histone methyltransferase SET Domain Bifurcated 1 (SETDB1). The same report proposed that H3K9me3 stimulates APB formation, telomeric recombination and TERRA transcription (Gauchier et al., 2019). Further studies are thus necessary to fully understand the intricate interplay between heterochromatin deposition and ALT establishment and/or maintenance.

ALT HDR occurs through Break-Induced Replication (BIR) in the G2/M phase of the cell cycle. BIR is a conservative DNA synthesis-based repair pathway engaging at one-ended DNA double-strand breaks (DSBs) and arrested replication forks (Kramara et al., 2018). Two types of BIR, either RAD51- or RAD52-dependent, were firstly identified in ALT yeasts (Le et al., 1999; Chen et al., 2001). In human ALT cells, BIR does not require RAD51 while it depends on RAD52 and on the DNA polymerase δ accessory subunits POLD3 and POLD4, and on PCNA (Roumelioti et al., 2016; Zhang et al., 2019). ALT dependence on telomeric HDR implies that at least a subset of telomeres is maintained physiologically damaged. This sustained damage is replication-dependent, explaining the constitutive association of replication stress-associated factors, such as RPA and its phosphorylation-modified versions, with ALT telomeres (Arora et al., 2014; Pan et al., 2017). Although the triggers of this ALT-specific Telomeric Replication Stress (herein referred to as ATRS) have not been fully elucidated, a variety of hypotheses can be envisaged. Telomeres are difficult to replicate regions because of the repetitive nature of their sequence, the tight association of telomeric DNA with heterochromatin marks and telomeric proteins, and their richness in higher order structures including T-loops, generated upon intramolecular invasion of the 3′ end ss tail of a telomere into its ds part, and telomeric R-loops (telR-loops), formed by annealing of TERRA with the C-rich strand of the telomere (Sfeir et al., 2009; Balk et al., 2013; Pfeiffer et al., 2013; Arora et al., 2014). Additionally, G-quadruplexes may form when the G-rich telomeric strand exists in ss state, for example at the displacement loop of a T-loop or a telR-loop (Tarsounas and Tijsterman, 2013). Improper handling of any of those features could contribute to ATRS.

Because replication stress impairs cell proliferation through activation of DNA damage checkpoints, alleviators of ATRS are constantly active in ALT cells. The endoribonuclease RNaseH1 associates with ALT telomeres, where it degrades the RNA moiety of telR-loops. Short interference RNA (siRNA)-mediated depletion of RNaseH1 in ALT cells increases telR-loops, ATRS and circular telomeric molecules comprising ss C-rich DNA (C-circles), and ultimately causes rapid loss of entire telomeric tracts (Arora et al., 2014). The ATP-dependent DNA annealing helicase SWI/SNF-related, Matrix-associated, Actin-dependent Regulator of Chromatin, subfamily A-Like 1 (SMARCAL1), which restarts arrested replication forks through fork regression, is enriched at ALT telomeres, and its depletion using siRNAs augments ATRS, telomeric DNA damage and ALT features including C-circles (Cox et al., 2016). The checkpoint kinase Ataxia Telangiectasia and Rad3-Related (ATR) is also found at ALT telomeres and its inactivation using siRNAs or small molecule inhibitors increases ATRS and leads to cell death specifically in ALT cells, although this last notion has been questioned (Flynn et al., 2015; Deeg et al., 2016).

## FANCM and ALT

FANCM involvement in ALT was first reported by the Zhang laboratory in 2017 (Pan et al., 2017). The authors showed that FANCM and FAAP24 localize to telomeres in a variety of ALT cell lines. SiRNA-mediated depletion of FANCM, FAAP24, MHF1, or MHF2 induced ATRS in ALT cells, as demonstrated by the telomeric localization of phosphorylated RPA and the checkpoint kinase CHK1 (Pan et al., 2017). Single Molecule Analysis of Replicated DNA (SMARD) using telomeric DNA from FANCM-depleted ALT cells revealed diminished replication efficiencies, while replication genome-wide was only minimally affected (Pan et al., 2017). Overall those data indicate that in absence of FANCM the replication machinery fails to fully replicate telomeric DNA, thus leading to ATRS. FANCM depletion was also shown to cause accumulation of BLM and BRCA1 at ALT telomeres, and simultaneous depletion of either of those factors together with FANCM partly averted ATRS (Pan et al., 2017). The authors proposed that ATRS induced by FANCM depletion promotes recruitment of BLM and BRCA1 to telomeres, where the two factors enhance end resection in order to restart arrested forks and repair telomeric DNA through HDR. Apparently consistent with this model, co-depletions of FANCM with BLM or BRCA1 were shown to be synthetically lethal, specifically in ALT cells (Pan et al., 2017).

Two recent reports, from the Pickett laboratory and ours, have deepened our understanding of how FANCM functions at ALT telomeres. Both reports confirmed that FANCM depletion in ALT cells causes ATRS and telomeric DNA damage. Accumulation of phosphorylated RPA, ssDNA, and the DNA damage marks γH2AX and 53BP1 was observed at telomeres in cells depleted of FANCM using independent siRNAs (Lu et al., 2019; Silva et al., 2019). The two reports also established that FANCM suppresses ALT activity, likely as a consequence of ATRS alleviation. Augmented ALT features, including telomere clustering in large APBs, C-circle production and POLD3-mediated telomeric BIR in G2, were observed in FANCM-depleted ALT cells. Conversely, ALT features were not detected in telomerase-positive cells depleted of FANCM, indicating that FANCM inhibition does not cause ALT but rather FANCM has acquired specific telomeric functions in cells with already established ALT activity (Lu et al., 2019; Silva et al., 2019). Moreover, both studies revealed that FANCM inhibition alone is extremely toxic for ALT cells, as it leads to rapid arrest of cell cycle progression in G2/M phase followed by cell death (Lu et al., 2019; Silva et al., 2019). FANCM essentiality for ALT cell viability was further confirmed by interrogating publicly available catalogs of CRISPR/Cas9 gene knock-outs across cancer cell lines (Lu et al., 2019). These observations are in contrast with previous work from Pan and colleagues (Pan et al., 2017), who showed that FANCM depletion alone is not detrimental in ALT cells. It is likely that different siRNA efficiencies and experimental set ups for cell proliferation analysis account for those discrepancies.

Mechanistic insights from those two recent reports highlight the complexity of the mechanisms orchestrated by FANCM in ALT cells. We showed that telR-loops accumulate when FANCM is depleted, and orthogonal resolution of telR-loops through over-expression of RNaseH1 alleviates FANCM depletion-induced ATRS. FANCM likely restricts telR-loops directly, because FANCM can unwind telR-loops *in vitro* in an ATP-dependent manner and the K117R mutant fails to avert ATRS in FANCM-depleted cells (Silva et al., 2019). Moreover, we confirmed that BLM depletion alleviates ATRS in FANCM-depleted cells, and showed that combined RNaseH1 over-expression and BLM depletion fully eliminates ATRS induced by FANCM depletion (Silva et al., 2019). We thus proposed that deregulated telR-loops and BLM are the main triggers of ATRS, consequent ALT exacerbation and cell death when FANCM activity is inhibited. It remains possible that, besides R-loops resolution, other functions associated with the ATPase activity of FANCM (Collis et al., 2008; Schwab et al., 2010; Huang et al., 2013; Wang et al., 2018) could help support telomere stability and viability in ALT cells.

On the other side, the report by Lu and colleagues focused on the importance of the interaction between FANCM and the BTR complex. They showed that over-expression of FANCM suppresses ALT features including damaged telomeres and C-circles. Conversely, over-expression of two mutant versions of FANCM unable to bind the BTR complex did not suppress those features (Lu et al., 2019). Consistent with our results, also the K117R mutant failed to suppress ALT, while a mutant unable to interact with the FA core complex behaved as wild-type FANCM when over-expressed (Lu et al., 2019). Altogether, this set of data confirms the importance of the enzymatic activity of FANCM in suppressing ALT, reveals the relevance of the interaction between FANCM and the BTR complex, and excludes that FANCM suppresses ALT as a member of the FA core complex. Moreover, the Pickett group utilized two independent approaches to prevent the interaction between FANCM and the BTR complex in cells: ectopic expression of a 28 aa long peptide from the MM2 sequence of FANCM or treatment with the small molecule inhibitor PIP-199. In both cases, FANCM-BTR interaction interference caused telomeric DNA damage and loss of cell viability specifically in ALT cells (Lu et al., 2019).

Collectively, the recent reports on FANCM in ALT established that FANCM is an alleviator of ATRS and unveiled two main co-players, telR-loops and BLM. TelR-loops are abundant at ALT telomeres and are kept in check by dedicated machineries including RNaseH1 and FANCM. Inactivation of such machineries induces telR-loop stabilization and ATRS (Arora et al., 2014; Silva et al., 2019). Although strongly suggesting that telR-loops are main triggers of ATRS, this evidence remains correlative, as systems to modulate TERRA transcription in cells are not available. It is now necessary to develop such systems and test the involvement of TERRA in telR-loop formation and ATRS. Moreover, while it is clear that RNaseH1 activity negatively regulates ATRS (Arora et al., 2014), the impact of its depletion or over-expression on the ALT mechanisms has not been fully tested. Analysis of ALT features including APBs and BIR in cells with altered RNaseH1 activity will help address this point.

**Figure 2:**
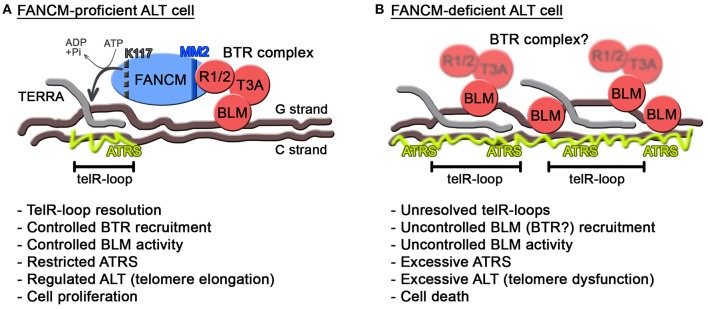
Model for FANCM function at ALT telomeres. **(A)** In FANCM-proficient ALT cells, FANCM association with telomeric chromatin assures unwinding of harmful telR-loops through its ATPase activity. Additionally, FANCM interaction with RMI1/2 assures regulated recruitment and activity of BLM. In this situation, ATRS is maintained below toxic levels allowing telomere elongation and infinite cell proliferation. In FANCM, lysine K117 and the MM2 motif are indicated by a dotted black line and a blue line, respectively. **(B)** In FANCM-deficient ALT cells, telR-loops accumulate and BLM is aberrantly recruited and activated, leading to excessive ATRS and eventually cell death. RMI1/2 and TOP3A are blurred to indicate that their recruitment to telomeres has not been tested yet in FANCM-deficient cells.

As for BLM, this helicase seems to have intimate yet intricate connections with ATRS, in particular in the context of FANCM deficiency. While on one side decreasing BLM levels alleviates ATRS when FANCM is depleted (Pan et al., 2017; Silva et al., 2019), replacement of endogenous FANCM with a mutant unable to associate with the BTR complex exacerbates ATRS and ALT (Lu et al., 2019). Moreover, depletion of FANCM provokes BLM accumulation at ALT telomeres (Pan et al., 2017; Silva et al., 2019). Those apparently contradictory data can be reconciled by postulating independent activities for BLM. We propose that BLM supports regulated and productive ALT activity as long as it is properly controlled, possibly as a member of the BTR complex (Figure 2A). Consistently, depletion of any of the BTR members suppresses ALT features (Sobinoff and Pickett, 2017). Proper regulation of BLM at ALT telomeres would therefore depend on the BTR interaction with FANCM MM2 domain (Figure 2A). In the absence of this regulation, for example when FANCM is depleted or is replaced by a BTR interaction mutant, BLM could be recruited to telomeres through FANCM-independent routes and become hyperactive and therefore toxic (Figure 2B). It will be interesting to test whether RMI1 and TOP3A are also recruited to telomeres in FANCM-depleted cells and whether BLM localization at FANCM-depleted telomeres depends on the BTR complex.

Lastly, FANCM seems to be an optimal target for anti-ALT cancer therapies because it is a non-essential factor in normal and telomerase-positive cells. SiRNA-mediated depletion of FANCM in a large panel of non-ALT cells does not lead to cell cycle arrest or death (Lu et al., 2019; Silva et al., 2019). Telomerase-positive human colorectal carcinoma HCT116 cells, mouse embryonic fibroblasts and chicken DT40 cells knocked-out for FANCM proliferate normally unless challenged with DNA damaging agents (Mosedale et al., 2005; Bakker et al., 2009; Huang et al., 2013). Individuals with FANCM mutations reach adulthood without major complications (Meetei et al., 2005; Catucci et al., 2018). Targeting FANCM, more specifically its enzymatic activity or its interaction with the BTR complex, holds the potential for a successful treatment of ALT cancers. One possible caveat comes from the observations that human FANCM mutants might develop cancer, likely telomerase-positive, late in life (Catucci et al., 2018; Nurmi et al., 2019; Schubert et al., 2019). Nevertheless, considering the fast and dramatic effects of FANCM depletion on ALT cells, we anticipate that transient FANCM inhibition should be sufficient to extirpate ALT in absence of secondary effects on patients.

## Author Contributions

BD-S, BS, and CA wrote, revised, and edited the manuscript.

### Conflict of Interest Statement

The authors declare that the research was conducted in the absence of any commercial or financial relationships that could be construed as a potential conflict of interest.

## References

[B1] Acharya S. Kaul Z. Gocha A. S. Martinez A. R. Harris J. Parvin J. D.. (2014). Association of BLM and BRCA1 during Telomere Maintenance in ALT Cells. PLoS ONE 9:e103819. 10.1371/journal.pone.0103819 25084169 PMC4118958

[B2] Apte M. S. Cooper J. P. (2017). Life and cancer without telomerase: ALT and other strategies for making sure ends (don't) meet. Crit. Rev. Biochem. Mol. Biol. 52, 57–73. 10.1080/10409238.2016.1260090 27892716 PMC6329634

[B3] Arora R. Lee Y. Wischnewski H. Brun C. M. Schwarz T. Azzalin C. M. (2014). RNaseH1 regulates TERRA-telomeric DNA hybrids and telomere maintenance in ALT tumour cells. Nat. Commun. 5:5220. 10.1038/ncomms6220 25330849 PMC4218956

[B4] Azzalin C. M. Lingner J. (2015). Telomere functions grounding on TERRA firma. Trends Cell. Biol. 25, 29–36. 10.1016/j.tcb.2014.08.007 25257515

[B5] Bailey S. M. Brenneman M. A. Goodwin E. H. (2004). Frequent recombination in telomeric DNA may extend the proliferative life of telomerase-negative cells. Nucleic Acids Res. 32, 3743–3751. 10.1093/nar/gkh691 15258249 PMC484178

[B6] Bakker S. T. Van De Vrugt H. J. Rooimans M. A. Oostra A. B. Steltenpool J. Delzenne-Goette E.. (2009). Fancm-deficient mice reveal unique features of Fanconi anemia complementation group M. Hum. Mol. Genet. 18, 3484–3495. 10.1093/hmg/ddp297 19561169

[B7] Balk B. Maicher A. Dees M. Klermund J. Luke-Glaser S. Bender K.. (2013). Telomeric RNA-DNA hybrids affect telomere-length dynamics and senescence. Nat. Struct. Mol. Biol. 20, 1199–1205. 10.1038/nsmb.2662 24013207

[B8] Bogliolo M. Bluteau D. Lespinasse J. Pujol R. Vasquez N. D'enghien C. D. (2018). Biallelic truncating FANCM mutations cause early-onset cancer but not Fanconi anemia. Genet. Med. 20, 458–463. 10.1038/gim.2017.124 28837157

[B9] Bryan T. M. Englezou A. Dalla-Pozza L. Dunham M. A. Reddel R. R. (1997). Evidence for an alternative mechanism for maintaining telomere length in human tumors and tumor-derived cell lines. Nat. Med. 3, 1271–1274. 10.1038/nm1197-1271 9359704

[B10] Bryan T. M. Englezou A. Gupta J. Bacchetti S. Reddel R. R. (1995). Telomere elongation in immortal human cells without detectable telomerase activity. EMBO J. 14, 4240–4248. 10.1002/j.1460-2075.1995.tb00098.x 7556065 PMC394507

[B11] Catucci I. Osorio A. Arver B. Neidhardt G. Bogliolo M. Zanardi F. (2018). Individuals with FANCM biallelic mutations do not develop Fanconi anemia, but show risk for breast cancer, chemotherapy toxicity and may display chromosome fragility. Genet. Med. 20, 452–457. 10.1038/gim.2017.123 28837162

[B12] Cesare A. J. Griffith J. D. (2004). Telomeric DNA in ALT cells is characterized by free telomeric circles and heterogeneous t-loops. Mol. Cell. Biol. 24, 9948–9957. 10.1128/MCB.24.22.9948-9957.2004 15509797 PMC525488

[B13] Chen Q. Ijpma A. Greider C. W. (2001). Two survivor pathways that allow growth in the absence of telomerase are generated by distinct telomere recombination events. Mol. Cell. Biol. 21, 1819–1827. 10.1128/MCB.21.5.1819-1827.2001 11238918 PMC86745

[B14] Ciccia A. Ling C. Coulthard R. Yan Z. Xue Y. Meetei A. R.. (2007). Identification of FAAP24, a Fanconi anemia core complex protein that interacts with FANCM. Mol. Cell. 25, 331–343. 10.1016/j.molcel.2007.01.003 17289582

[B15] Collis S. J. Ciccia A. Deans A. J. Horejsi Z. Martin J. S. Maslen S. L.. (2008). FANCM and FAAP24 function in ATR-mediated checkpoint signaling independently of the Fanconi anemia core complex. Mol. Cell. 32, 313–324. 10.1016/j.molcel.2008.10.014 18995830

[B16] Coulthard R. Deans A. J. Swuec P. Bowles M. Costa A. West S. C.. (2013). Architecture and DNA recognition elements of the Fanconi anemia FANCM-FAAP24 complex. Structure 21, 1648–1658. 10.1016/j.str.2013.07.006 23932590 PMC3763369

[B17] Cox K. E. Marechal A. Flynn R. L. (2016). SMARCAL1 Resolves Replication Stress at ALT Telomeres. Cell Rep. 14, 1032–1040. 10.1016/j.celrep.2016.01.011 26832416 PMC5051350

[B18] Crossley M. P. Bocek M. Cimprich K. A. (2019). R-loops as cellular regulators and genomic threats. Mol. Cell. 73, 398–411. 10.1016/j.molcel.2019.01.024 30735654 PMC6402819

[B19] Deans A. J. West S. C. (2009). FANCM connects the genome instability disorders Bloom's Syndrome and Fanconi Anemia. Mol. Cell. 36, 943–953. 10.1016/j.molcel.2009.12.006 20064461

[B20] Deeg K. I. Chung I. Bauer C. Rippe K. (2016). Cancer cells with alternative lengthening of telomeres do not display a general hypersensitivity to ATR inhibition. Front. Oncol. 6:186 10.3389/fonc.2016.00186 27602331 PMC4993795

[B21] Dunham M. A. Neumann A. A. Fasching C. L. Reddel R. R. (2000). Telomere maintenance by recombination in human cells. Nat. Genet. 26, 447–450. 10.1038/82586 11101843

[B22] Dyer M. A. Qadeer Z. A. Valle-Garcia D. Bernstein E. (2017). ATRX and DAXX: mechanisms and mutations. Cold Spring Harb. Perspect. Med. 7:a026567. 10.1101/cshperspect.a026567 28062559 PMC5334245

[B23] Episkopou H. Draskovic I. Van Beneden A. Tilman G. Mattiussi M. Gobin M.. (2014). Alternative Lengthening of Telomeres is characterized by reduced compaction of telomeric chromatin. Nucleic Acids Res. 42, 4391–4405. 10.1093/nar/gku114 24500201 PMC3985679

[B24] Flynn R. L. Cox K. E. Jeitany M. Wakimoto H. Bryll A. R. Ganem N. J.. (2015). Alternative lengthening of telomeres renders cancer cells hypersensitive to ATR inhibitors. Science 347, 273–277. 10.1126/science.1257216 25593184 PMC4358324

[B25] Garcia-Rubio M. L. Perez-Calero C. Barroso S. I. Tumini E. Herrera-Moyano E. Rosado I. V.. (2015). The fanconi anemia pathway protects genome integrity from R-loops. PLoS Genet. 11:e1005674. 10.1371/journal.pgen.1005674 26584049 PMC4652862

[B26] Gauchier M. Kan S. Barral A. Sauzet S. Agirre E. Bonnell E.. (2019). SETDB1-dependent heterochromatin stimulates alternative lengthening of telomeres. Sci. Adv. 5:eaav3673. 10.1126/sciadv.aav3673 31086817 PMC6506250

[B27] Harley C. B. Futcher A. B. Greider C. W. (1990). Telomeres shorten during ageing of human fibroblasts. Nature 345, 458–460. 10.1038/345458a0 2342578

[B28] Heaphy C. M. De Wilde R. F. Jiao Y. Klein A. P. Edil B. H. Shi C.. (2011). Altered telomeres in tumors with ATRX and DAXX mutations. Science 333:425. 10.1126/science.1207313 21719641 PMC3174141

[B29] Hoadley K. A. Xue Y. Ling C. Takata M. Wang W. Keck J. L. (2012). Defining the molecular interface that connects the Fanconi anemia protein FANCM to the Bloom syndrome dissolvasome. Proc. Natl. Acad. Sci. U.S.A. 109, 4437–4442. 10.1073/pnas.1117279109 22392978 PMC3311393

[B30] Huang J. Liu S. Bellani M. A. Thazhathveetil A. K. Ling C. De Winter J. P.. (2013). The DNA translocase FANCM/MHF promotes replication traverse of DNA interstrand crosslinks. Mol. Cell. 52, 434–446. 10.1016/j.molcel.2013.09.021 24207054 PMC3880019

[B31] Johnson F. B. Marciniak R. A. Mcvey M. Stewart S. A. Hahn W. C. Guarente L. (2001). The Saccharomyces cerevisiae WRN homolog Sgs1p participates in telomere maintenance in cells lacking telomerase. EMBO J. 20, 905–913. 10.1093/emboj/20.4.905 11179234 PMC145415

[B32] Karow J. K. Constantinou A. Li J. L. West S. C. Hickson I. D. (2000). The Bloom's syndrome gene product promotes branch migration of holliday junctions. Proc. Natl. Acad. Sci. U.S.A. 97, 6504–6508. 10.1073/pnas.100448097 10823897 PMC18638

[B33] Kim N. W. Piatyszek M. A. Prowse K. R. Harley C. B. West M. D. Ho P. L.. (1994). Specific association of human telomerase activity with immortal cells and cancer. Science 266, 2011–2015. 10.1126/science.7605428 7605428

[B34] Klein Douwel D. Boonen R. A. Long D. T. Szypowska A. A. Raschle M. Walter J. C.. (2014). XPF-ERCC1 acts in Unhooking DNA interstrand crosslinks in cooperation with FANCD2 and FANCP/SLX4. Mol. Cell. 54, 460–471. 10.1016/j.molcel.2014.03.015 24726325 PMC5067070

[B35] Kramara J. Osia B. Malkova A. (2018). Break-induced replication: the where, the why, and the how. Trends Genet. 34, 518–531. 10.1016/j.tig.2018.04.002 29735283 PMC6469874

[B36] Lafuente-Barquero J. Luke-Glaser S. Graf M. Silva S. Gomez-Gonzalez B. Lockhart A.. (2017). The Smc5/6 complex regulates the yeast Mph1 helicase at RNA-DNA hybrid-mediated DNA damage. PLoS Genet. 13:e1007136. 10.1371/journal.pgen.1007136 29281624 PMC5760084

[B37] Le S. Moore J. K. Haber J. E. Greider C. W. (1999). RAD50 and RAD51 define two pathways that collaborate to maintain telomeres in the absence of telomerase. Genetics. 152, 143–152. 10224249 10.1093/genetics/152.1.143 PMC1460580

[B38] Lovejoy C. A. Li W. Reisenweber S. Thongthip S. Bruno J. De Lange T.. (2012). Loss of ATRX, genome instability, and an altered DNA damage response are hallmarks of the alternative lengthening of telomeres pathway. PLoS Genet. 8:e1002772. 10.1371/journal.pgen.1002772 22829774 PMC3400581

[B39] Lu R. O'rourke J. J. Sobinoff A. P. Allen J. A. M Nelson C. B. Pickett H. A.. (2019). The FANCM-BLM-TOP3A-RMI complex suppresses alternative lengthening of telomeres (ALT). Nat. Commun. 10:2252. 10.1038/s41467-019-10180-6 31138797 PMC6538672

[B40] Lundblad V. Blackburn E. H. (1993). An alternative pathway for yeast telomere maintenance rescues est1- senescence. Cell 73, 347–360. 10.1016/0092-8674(93)90234-H 8477448

[B41] Meetei A. R. Medhurst A. L. Ling C. Xue Y. Singh T. R. Bier P.. (2005). A human ortholog of archaeal DNA repair protein Hef is defective in Fanconi anemia complementation group M. Nat. Genet. 37, 958–963. 10.1038/ng1626 16116422 PMC2704909

[B42] Mosedale G. Niedzwiedz W. Alpi A. Perrina F. Pereira-Leal J. B. Johnson M.. (2005). The vertebrate Hef ortholog is a component of the Fanconi anemia tumor-suppressor pathway. Nat. Struct. Mol. Biol. 12, 763–771. 10.1038/nsmb981 16116434

[B43] Nassour J. Radford R. Correia A. Fuste J. M. Schoell B. Jauch A.. (2019). Autophagic cell death restricts chromosomal instability during replicative crisis. Nature 565, 659–663. 10.1038/s41586-019-0885-0 30675059 PMC6557118

[B44] Neff N. F. Ellis N. A. Ye T. Z. Noonan J. Huang K. Sanz M.. (1999). The DNA helicase activity of BLM is necessary for the correction of the genomic instability of bloom syndrome cells. Mol. Biol. Cell. 10, 665–676. 10.1091/mbc.10.3.665 10069810 PMC25194

[B45] Nurmi A. Muranen T. A. Pelttari L. M. Kiiski J. I. Heikkinen T. Lehto S.. (2019). Recurrent moderate-risk mutations in Finnish breast and ovarian cancer patients. Int. J. Cancer. [Epub ahead of print]. 10.1002/ijc.32309 30927251 PMC6767104

[B46] Ogino H. Nakabayashi K. Suzuki M. Takahashi E. Fujii M. Suzuki T.. (1998). Release of telomeric DNA from chromosomes in immortal human cells lacking telomerase activity. Biochem. Biophys. Res. Commun. 248, 223–227. 10.1006/bbrc.1998.8875 9675117

[B47] Pan X. Drosopoulos W. C. Sethi L. Madireddy A. Schildkraut C. L. Zhang D. (2017). FANCM, BRCA1, and BLM cooperatively resolve the replication stress at the ALT telomeres. Proc. Natl. Acad. Sci. U.S.A. 114, E5940–E5949. 10.1073/pnas.1708065114 28673972 PMC5530707

[B48] Pfeiffer V. Crittin J. Grolimund L. Lingner J. (2013). The THO complex component Thp2 counteracts telomeric R-loops and telomere shortening. EMBO J. 32, 2861–2871. 10.1038/emboj.2013.217 24084588 PMC3817467

[B49] Rohleder F. Huang J. Xue Y. Kuper J. Round A. Seidman M.. (2016). FANCM interacts with PCNA to promote replication traverse of DNA interstrand crosslinks. Nucleic Acids Res 44, 3219–3232. 10.1093/nar/gkw037 26825464 PMC4838364

[B50] Roumelioti F. M. Sotiriou S. K. Katsini V. Chiourea M. Halazonetis T. D. Gagos S. (2016). Alternative lengthening of human telomeres is a conservative DNA replication process with features of break-induced replication. EMBO Rep. 17, 1731–1737. 10.15252/embr.201643169 27760777 PMC5167343

[B51] Schubert S. Van Luttikhuizen J. L. Auber B. Schmidt G. Hofmann W. Penkert J.. (2019). The identification of pathogenic variants in BRCA1/2 negative, high risk, hereditary breast and/or ovarian cancer patients: high frequency of FANCM pathogenic variants. Int. J. Cancer 144, 2683–2694. 10.1002/ijc.31992 30426508

[B52] Schwab R. A. Blackford A. N. Niedzwiedz W. (2010). ATR activation and replication fork restart are defective in FANCM-deficient cells. EMBO J. 29, 806–818. 10.1038/emboj.2009.385 20057355 PMC2829160

[B53] Schwab R. A. Nieminuszczy J. Shah F. Langton J. Lopez Martinez D. Liang C. C.. (2015). The fanconi anemia pathway maintains genome stability by coordinating replication and transcription. Mol. Cell. 60, 351–361. 10.1016/j.molcel.2015.09.012 26593718 PMC4644232

[B54] Schwartzentruber J. Korshunov A. Liu X. Y. Jones D. T. Pfaff E. Jacob K.. (2012). Driver mutations in histone H3.3 and chromatin remodelling genes in paediatric glioblastoma. Nature 482, 226–231. 10.1038/nature10833 22286061

[B55] Sfeir A. Kosiyatrakul S. T. Hockemeyer D. Macrae S. L. Karlseder J. Schildkraut C. L.. (2009). Mammalian telomeres resemble fragile sites and require TRF1 for efficient replication. Cell 138, 90–103. 10.1016/j.cell.2009.06.021 19596237 PMC2723738

[B56] Shay J. W. Bacchetti S. (1997). A survey of telomerase activity in human cancer. Eur. J. Cancer 33, 787–791. 10.1016/S0959-8049(97)00062-2 9282118

[B57] Shay J. W. Wright W. E. (2019). Telomeres and telomerase: three decades of progress. Nat. Rev. Genet. 20, 299–309. 10.1038/s41576-019-0099-1 30760854

[B58] Silva B. Pentz R. Figueira A. M. Arora R. Lee Y. W. Hodson C.. (2019). FANCM limits ALT activity by restricting telomeric replication stress induced by deregulated BLM and R-loops. Nat. Commun. 10:2253. 10.1038/s41467-019-10179-z 31138795 PMC6538666

[B59] Singh T. R. Bakker S. T. Agarwal S. Jansen M. Grassman E. Godthelp B. C.. (2009). Impaired FANCD2 monoubiquitination and hypersensitivity to camptothecin uniquely characterize Fanconi anemia complementation group M. Blood 114, 174–180. 10.1182/blood-2009-02-207811 19423727 PMC2710946

[B60] Sobinoff A. P. Pickett H. A. (2017). Alternative lengthening of telomeres: DNA repair pathways converge. Trends Genet. 33, 921–932. 10.1016/j.tig.2017.09.003 28969871

[B61] Stavropoulos D. J. Bradshaw P. S. Li X. Pasic I. Truong K. Ikura M.. (2002). The Bloom syndrome helicase BLM interacts with TRF2 in ALT cells and promotes telomeric DNA synthesis. Hum. Mol. Genet. 11, 3135–3144. 10.1093/hmg/11.25.3135 12444098

[B62] Tarsounas M. Tijsterman M. (2013). Genomes and G-quadruplexes: for better or for worse. J. Mol. Biol. 425, 4782–4789. 10.1016/j.jmb.2013.09.026 24076189

[B63] Tokutake Y. Matsumoto T. Watanabe T. Maeda S. Tahara H. Sakamoto S.. (1998). Extra-chromosomal telomere repeat DNA in telomerase-negative immortalized cell lines. Biochem. Biophys. Res. Commun. 247, 765–772. 10.1006/bbrc.1998.8876 9647768

[B64] Wang H. Li S. Oaks J. Ren J. Li L. Wu X. (2018). The concerted roles of FANCM and Rad52 in the protection of common fragile sites. Nat. Commun. 9:2791. 10.1038/s41467-018-05066-y 30022024 PMC6052092

[B65] Wang R. C. Smogorzewska A. De Lange T. (2004). Homologous recombination generates T-loop-sized deletions at human telomeres. Cell 119, 355–368. 10.1016/j.cell.2004.10.011 15507207

[B66] Wu L. Hickson I. D. (2003). The Bloom's syndrome helicase suppresses crossing over during homologous recombination. Nature 426, 870–874. 10.1038/nature02253 14685245

[B67] Xue Y. Li Y. Guo R. Ling C. Wang W. (2008). FANCM of the Fanconi anemia core complex is required for both monoubiquitination and DNA repair. Hum. Mol. Genet. 17, 1641–1652. 10.1093/hmg/ddn054 18285517 PMC2902294

[B68] Yamamoto K. N. Kobayashi S. Tsuda M. Kurumizaka H. Takata M. Kono K.. (2011). Involvement of SLX4 in interstrand cross-link repair is regulated by the Fanconi anemia pathway. Proc. Natl. Acad. Sci. U.S.A. 108, 6492–6496. 10.1073/pnas.1018487108 21464321 PMC3080998

[B69] Yan Z. Delannoy M. Ling C. Daee D. Osman F. Muniandy P. A.. (2010). A histone-fold complex and FANCM form a conserved DNA-remodeling complex to maintain genome stability. Mol. Cell. 37, 865–878. 10.1016/j.molcel.2010.01.039 20347428 PMC2847587

[B70] Yang H. Zhang T. Tao Y. Wang F. Tong L. Ding J. (2013). Structural insights into the functions of the FANCM-FAAP24 complex in DNA repair. Nucleic Acids Res. 41, 10573–10583. 10.1093/nar/gkt788 24003026 PMC3905867

[B71] Yeager T. R. Neumann A. A. Englezou A. Huschtscha L. I. Noble J. R. Reddel R. R. (1999). Telomerase-negative immortalized human cells contain a novel type of promyelocytic leukemia (PML) body. Cancer Res. 59, 4175–4179. 10485449

[B72] Zhang J. M. Yadav T. Ouyang J. Lan L. Zou L. (2019). Alternative lengthening of telomeres through two distinct break-induced replication pathways. Cell Rep. 26, 955–968 e953. 10.1016/j.celrep.2018.12.102 30673617 PMC6366628

